# Global Cancer Inequalities

**DOI:** 10.3389/fonc.2018.00293

**Published:** 2018-08-14

**Authors:** Miranda M. Fidler, Freddie Bray

**Affiliations:** Section of Cancer Surveillance, International Agency for Research on Cancer, Lyon, France

**Keywords:** social inequalities, cancer, global, incidence, mortality, DALYs

## Abstract

Social inequalities in cancer are increasingly relevant to research, implementation science, and policy. In this brief perspective we provide an overview of global cancer inequalities by assessing different outcomes according to the Human Development Index (HDI); the HDI is a United Nations Development Programme composite indicator including the following measures: (i) access to education (based on mean and expected years of schooling), (ii) a long and healthy life (based on life expectancy), and (iii) a decent standard of living (based on gross national income per capita). We additionally touch upon the importance of prevention, access to oncological services, and the need to monitor progress in reducing and avoiding inequalities at subnational, national, world region, and global levels.

Although social inequalities are well documented in cancer at macro and micro levels (1), their strong and persistent presence across the cancer continuum is increasingly relevant to research, implementation science, and policy. Cancer disparities research has moved beyond macroeconomic analyses involving comparative assessments of socioeconomic status or national income to investigate the multiple determinants of social inequality, exemplified through the assessment of demographic factors, including gender, age, race and ethnicity, and indigenous status. The enquiry as to how the global cancer burden (incidence, mortality, survival, disability-adjusted life years) is impacted by inequalities in socioeconomic development has moved on too. The exploration of the scale and profile of cancer, once set against the time-honored dichotomy of populations being “developing” or “developed,” has been superseded by the four-tier Human Development Index (HDI) (2), a broad marker of inequality and cancer transition between countries (3, 4).

The HDI asserts that an assessment of development is not just about economic growth but rather how national policies impact on human choices. The indicator is a composite measure incorporating (i) access to education (based on mean and expected years of schooling), (ii) a long and healthy life (based on life expectancy), and (iii) a decent standard of living (based on gross national income per capita) (2). It is commonly presented, as it is here, according to United Nations Development Programme's pre-defined cut-points representing four tiers of the HDI (from low HDI, e.g., nations with lowest human development values, through to medium, high, and very high, e.g., those with highest human development values).

Using this criterion, a strong correlation between the magnitude of the overall cancer incidence and the corresponding HDI level can be observed, with the overall cancer rates broadly increasing with level of human development (4). When assessed by specific cancers, positive relationships between the HDI level and incidence rate in 2012 were observed in both sexes for the following cancers: brain/nervous system, colorectum, gallbladder, kidney, leukemia, lung, multiple myeloma, pancreas, and thyroid; a positive relationship was also observed for bladder, lip/oral cavity, other pharyngeal, and testicular cancers, Hodgkin lymphoma, and melanoma in males, and breast, corpus uteri, and ovarian cancers and non-Hodgkin lymphoma in females (4). A negative association

between the HDI level and incidence rate was observed for cervical and other pharyngeal cancers and Kaposi sarcoma in females (4).

In terms of cancer profiles, the distribution of common cancers is quite different by HDI level, with infection- and poverty-related cancers (e.g., cervical and liver cancer) still dominating in low HDI nations, in contrast to high and very high HDI countries, where prostate, breast, colorectal, and lung are the major cancers (Figure 1). An increased burden of infection-related cancers with lower HDI is highlighted when one examines the population fractions of cancers attributable to infectious agents, which were estimated to be 25, 22, 13, and 8% in low, medium, high, and very high HDI countries, respectively, in 2012 (5).

**Figure 1:**
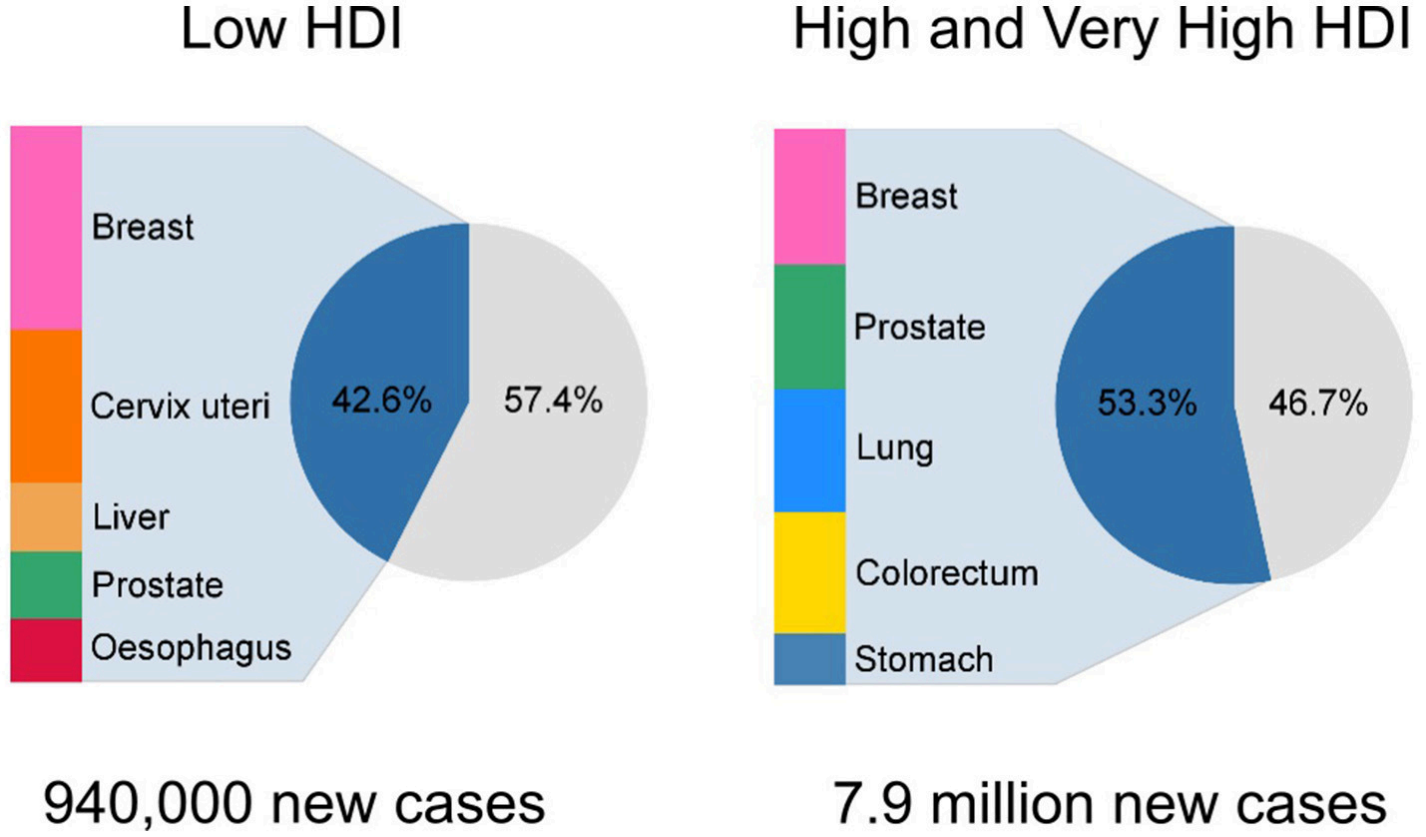
Number of new incidence cases and proportion of top five cancers in for the low Human Development Index (HDI) and high/very high HDI level as estimated in GLOBOCAN 2012.

Other inequalities in the global cancer burden have also been noted when assessed by the HDI, including mortality (6), disability-adjusted life years (7), and relative gains in life expectancy (8). An important additional marker of cancer inequality pertains to HDI stratifications of the future cancer incidence burden, which reveals that the number of new cancer cases in future years will be proportionally greatest in lower HDI settings (Figure 2), with low and medium HDI countries projected to see a 112 and 86% respective increase in their incidence burden from 2012 to 2035. Hence the nations currently least equipped to deal with a pending increase in the number of cancer patients year-on-year will be most impacted.

**Figure 2:**
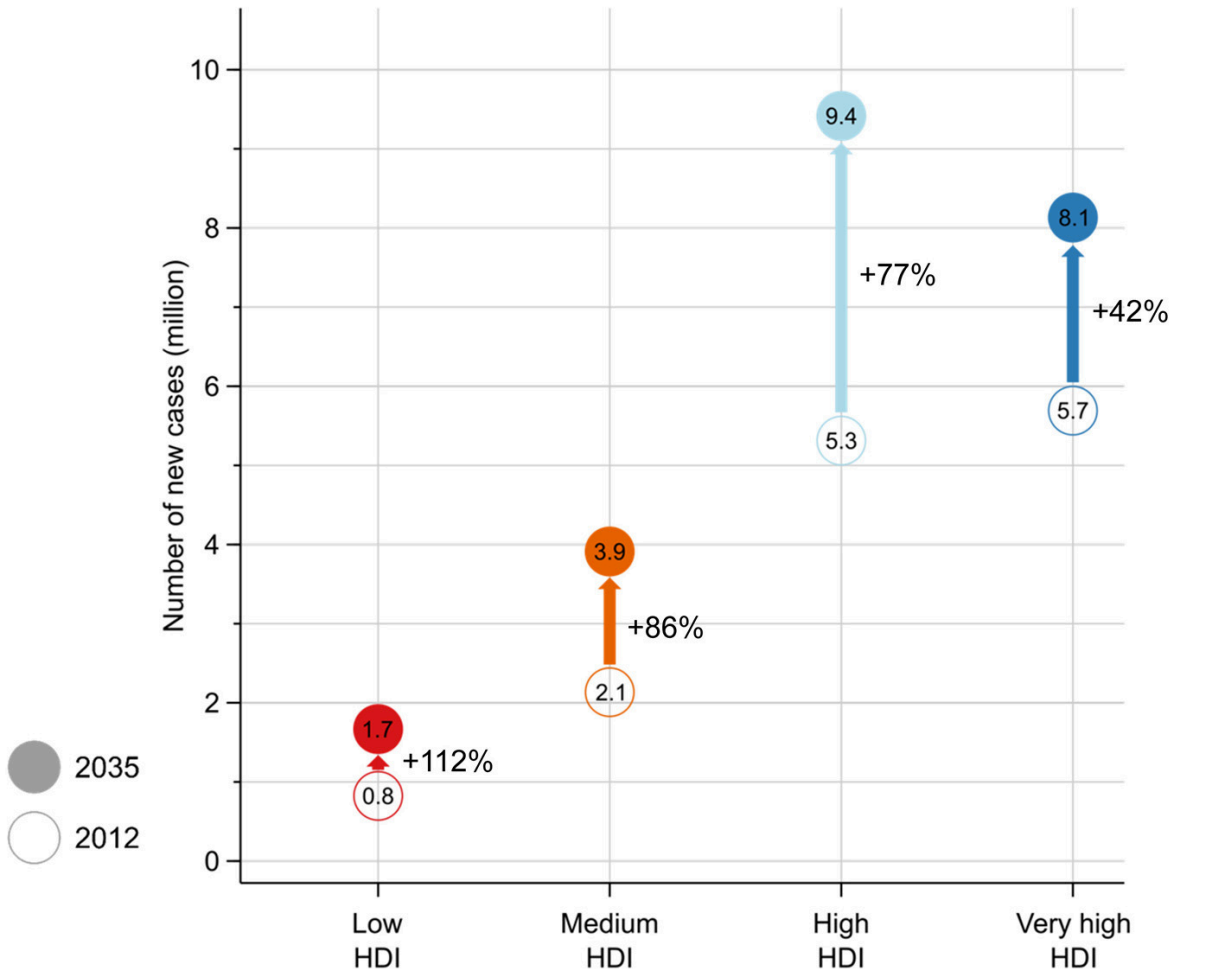
Number of estimated new cancer cases in 2012 and 2035 by the four-tier Human Development Index (HDI), with the proportional increase (%) in the number of cases indicated.

Such inequalities can only be expected to widen unless resource-dependant, effective, and cost-effective interventions are urgently implemented (9, 10). In particular, vaccination will be a key preventive strategy in low HDI settings given the high burden of infection-related cancers (5). Among the most important infections associated with cancer are hepatitis B virus and human papillomavirus, both of which have highly effective vaccines to prevent liver cancer and cervical, anal, vulvar, vaginal, and penial cancers, respectively (11). Tobacco control is another main priority for cancer control in transitioning countries given the number of smokers is projected to increase in these countries (12, 13). Finally, as social and economic transition increases the prevalence of sedentary jobs, urban living, and high caloric intake, an opportunity for prevention exists for less developed countries to avoid known adverse lifestyle risk factors like obesity, low physical activity, and higher alcohol intake, which cause many of the cancers commonly seen in the most developed countries.

With a growing cancer burden, access to appropriate, affordable, and equitable treatment will also be crucial in lower HDI settings, especially as the current availability of essential cancer medicines (14), cancer surgery (15), and radiotherapy facilities (16) is sparse. Preventing exposures to risk factors, early detection, effective treatment, and palliative care requires support and resources, however. More broadly, governments from around the world adopted a Cancer Resolution at the World Health Assembly in 2017, building on the United Nations Sustainable Development Goals 2030 (SDG), and the SDG 3.4 target of a reduction of the premature mortality from non-communicable diseases by one-third by 2030. Prevention and early detection are given prominence in the Resolution, with an emphasis on tobacco control policies within the World Health Organization Framework Convention on Tobacco Control, as well as affordable and feasible vaccine and screening programs. Furthermore, measuring the cancer burden to inform planning, through the development of population-based cancer registries, is also given central importance.

In summary, the HDI provides a useful framework to map out the continuing transitions in cancer globally, and highlights the clear reality of increasing inequalities in countries presently indexed at lower levels of the HDI. That said, there remains a need for an integration of social indicators in cancer research across the continuum, as well as the use of innovative methodologies, in order to monitor progress in reducing and avoiding inequalities at subnational, national, world region, and global levels. This is exemplified by the fact that governments have acknowledged the presence of social inequalities in cancer in the Cancer Resolution, which notes that “…certain population groups experience inequalities in risk factor exposure and in access to screening, early diagnosis and timely and appropriate treatment, and that they also experience poorer outcomes for cancer,” and recommends including measurements of inequalities in the collection of high-quality population-based incidence and mortality data on cancer.

As cancer is emerging as the leading cause of premature death, given ongoing displacements of deaths from infection and parasitic diseases in the lower HDI spectrum and cardiovascular diseases in decline at the higher end (8), the development and implementation of operational cancer control plans that include feasible, affordable, and sustainable interventions is now imperative worldwide, and most markedly in countries undergoing major social and economic transition. Such efforts can therefore be seen not only as an effort to reduce the widening gaps in cancer inequality, but also as a means to decrease inequalities across the spectrum of causes.

## Data source availability

GLOBOCAN 2012 data is freely available for download at http://globocan.iarc.fr/Default.aspx. Human Development Index data is freely available for download at http://hdr.undp.org/en/data.

## Author contributions

MF drafted the article, FB revised the article, MF and FB agreed to submission.

### Conflict of interest statement

The authors declare that the research was conducted in the absence of any commercial or financial relationships that could be construed as a potential conflict of interest.
